# Differentiated vulvar intraepithelial neoplasia (dVIN): the most helpful histological features and the utility of cytokeratins 13 and 17

**DOI:** 10.1007/s00428-018-2436-8

**Published:** 2018-09-06

**Authors:** Shatavisha Dasgupta, Patricia C. Ewing-Graham, Folkert J. van Kemenade, Helena C. van Doorn, Vincent Noordhoek Hegt, Senada Koljenović

**Affiliations:** 1 Department of Pathology, Erasmus MC, University Medical Centre Rotterdam, Rotterdam, The Netherlands; 2 Department of Gynaecologic Oncology, Erasmus MC, University Medical Centre Rotterdam, Rotterdam, The Netherlands

**Keywords:** Differentiated vulvar intraepithelial neoplasia, Cytokeratin 13, Cytokeratin 17, Vulva, Histology, Immunohistochemistry

## Abstract

Differentiated vulvar intraepithelial neoplasia (dVIN) is the precursor lesion of HPV-negative vulvar squamous cell carcinoma (VSCC). The histopathological diagnosis of dVIN can be challenging, as it often resembles vulvar non-neoplastic epithelial disorders (NNED), especially lichen sclerosus (LS). We aimed to establish the most specific and reproducible histological features of dVIN and assessed cytokeratin 13 (CK13) and cytokeratin 17 (CK17) immunohistochemistry as a diagnostic aid. Consecutive cases of dVIN (*n* = 180) and LS (*n* = 105) from the period 2010 to 2013 were reviewed using a checklist of histological features. Each feature was recorded as ‘present’ or ‘absent’ and statistical comparison (dVIN vs LS) was made. Interobserver agreement between two pairs of pathologists was assessed for a subset of cases of dVIN (*n* = 31) and LS and other NNED (*n* = 23). Immunohistochemistry with CK13, CK17, MIB1 and p53 was performed on dVIN, LS, and other NNED cases. Macronucleoli, features of disturbed maturation and angulated nuclei were significantly more common in dVIN than LS (*p* < 0.001). We found ‘substantial agreement’ for the diagnosis of dVIN (*κ* = 0.71). Macronucleoli and deep keratinisation had the highest agreement. In dVIN, the mean percentage of cells staining with CK13 was 15 and with CK17, this was 74. For LS, the mean percentage of cells staining with CK13 was 31, and with CK17, this was 41. By plotting receiver operating characteristic curves (ROC), an area under the curve (AUC) of 0.52 was obtained for CK13, and an AUC of 0.87 was obtained for CK17. The most helpful histological features for diagnosing dVIN were macronucleoli, features of disturbed maturation, and angulated nuclei. Increased CK17 expression may have promise for supporting dVIN diagnosis.

**Electronic supplementary material:**

The online version of this article (10.1007/s00428-018-2436-8) contains supplementary material, which is available to authorized users.

## Introduction

Vulvar intraepithelial neoplasia (VIN) is widely accepted as the precursor lesion of vulvar squamous cell carcinoma (VSCC) [1]. VSCC arises via either a human papilloma virus (HPV)-associated pathway, or more commonly, via a mechanism independent of HPV, often being linked to chronic inflammatory conditions such as lichen sclerosus (LS) [1, 2]. Accordingly, two distinct subtypes of VIN are recognised: the HPV-associated high-grade squamous intraepithelial lesion/usual VIN (HSIL/uVIN) and the non-HPV-associated differentiated VIN (dVIN) [1]. HSIL is clinically identified by its multifocal, warty appearance and on histology by conspicuous cytological and architectural atypia [2]. Differentiated VIN, on the other hand, often produces ill-defined lesions, and on histology, notoriously mimics non-neoplastic epithelial disorders (NNED), particularly LS [1, 2]. As a result, dVIN is rarely (< 5% cases) identified in advance of a diagnosis of invasive malignancy, despite being the precursor lesion of the majority of VSCC [3]. Moreover, there is substantial interobserver variability in the histological diagnosis of dVIN [4, 5]. In a recent study amongst vulva pathology experts, ‘basal layer atypia’ was the only criterion that met consensus to be ‘essential’ for dVIN diagnosis [6]. However, even this feature may not be readily appreciable in every case. The histological features of dVIN have been extensively described in the literature, but they have not been quantified so far [2, 5, 7].

In order to aid this difficult histological diagnosis, immunohistochemical markers p53 and MIB1 are commonly used, but both have limitations for making the distinction from NNED [2]. Increased p53 staining (overexpression) in the basal and parabasal layers is seen in dVIN, as a reflection of missense mutations of the *TP53* gene [2]. Additionally, 25–30% cases of dVIN show complete absence of p53 staining (null pattern), due to nonsense mutations and deletions [8]. However, p53 overexpression also occurs in long-standing LS and squamous hyperplasia, albeit as a consequence of ischemic stress [9–13]. The proliferation marker MIB1 can be increased in dVIN, as well as in NNED [14].

Recently, the diagnostic utility of the immunohistochemical markers cytokeratin 13 (CK13) and cytokeratin 17 (CK17) has been established for oral epithelial dysplasia [15–17]. Loss of CK13 along with expression of CK17 has been reported in (high-grade) oral epithelial dysplasia [15–17]. Increased expression of CK17 has been reported for dVIN [14], but CK13 has not yet been explored for this lesion.

Through this study, we aimed to establish the histological features of dVIN, which are most helpful to reliably distinguish dVIN from LS. The immunohistochemical markers, CK13 and CK17, were evaluated as diagnostic adjuncts for dVIN. To the best of our knowledge, this is the first study to quantify the histological features of dVIN, and to assess both CK13 and CK17 for dVIN diagnosis.

## Materials and methods

Consecutive cases with a histological diagnosis of dVIN, LS, other NNED (e.g. lichen simplex chronicus, lichenoid inflammation, chronic non-specific inflammation, epithelial hyperplasia, hyperkeratosis) and VSCC, from the period 2010 to 2013 were identified from the electronic database of the Department of Pathology, Erasmus MC. All the data were anonymised. The slides of these cases were retrieved from the archives.

Our study comprised three steps: histological evaluation, reproducibility analysis and assessment of immunohistochemistry. All the cases of dVIN and LS that were identified were included for the histological evaluation, and subsets of dVIN, LS and NNED cases were used for the reproducibility and immunohistochemistry analyses. The details of each step are further elaborated below.

### Histological evaluation

Histological evaluation was conducted on dVIN and LS, which is the closest and most difficult differential of dVIN. We formulated a checklist of histological features for dVIN based on the literature [4, 5, 7]. The components of the checklist are listed and described below. For all cases of dVIN and LS, each of the features on the checklist was recorded as ‘present’ or ‘absent’. The statistical significance of each feature for the diagnosis of dVIN over LS was calculated.

#### Nuclear atypia

This included variation in nuclear size and shape, including angulated nuclei; abnormality of the nuclear chromatin, i.e. hyperchromatic or open chromatin; presence of macronucleoli, i.e. nucleoli visible at ×100 magnification and multinucleation.

#### Mitoses

The presence of suprabasal and/or atypical mitotic figures was noted. The number of mitotic figures per 5 mm of the epithelium was counted.

#### Disturbed maturation

Disturbed maturation leads to premature keratinisation in the deeper layers of the epithelium, which was identified by a hyper-eosinophilic appearance. Individual cell keratinisation, deep keratinisation and deep squamous eddies (abortive pearls of keratin) were recorded as hallmarks of premature keratinisation. Cobblestone appearance of the epithelium, which is a combination of premature keratinisation with spongiosis, was recorded.

#### Architecture

Elongated (and anastomosing) rete ridges were noted.

#### Other features

Hyperkeratosis, parakeratosis, sub-epithelial hyalinisation and inflammatory cell infiltration were also recorded.

### Reproducibility

A representative subset of 54 cases were selected by two pathologists, SDG and PEG. The set comprised 31 dVIN, 10 LS and 13 other NNED cases. The cases were deliberately selected to provide a range of challenges. Thus, dVIN with classical histological appearances, as well as dVIN with ambiguous features, i.e. where the distinction between dVIN and LS was more difficult, were included. Glass slides of these cases were independently assessed by two other pathologists, SK and VNH. They were asked to provide a diagnosis for each case and adjudge the usefulness of the histological features for their diagnosis. The clinical history of the cases was not provided. No consensus training preceded the study. The agreement between the pathologists for (i) the overall diagnosis and (ii) the presence of the individual histological features identified as most specific (from the checklist described above) was measured.

### Immunohistochemistry

Immunohistochemistry was conducted on a subset of cases of dVIN, LS, and NNED. This set included cases found to have a good agreement for their diagnoses amongst pathologists in the reproducibility study and additional ones.

Sections of 4-μ thickness were prepared from formalin-fixed paraffin-embedded (FFPE) tissue. Cytokeratin 13 (clone KS-1A3, dilution 1:400, ThermoFisher); dual stain CK17-MIB1 (clone SP-95, ready to use, Ventana) and p53 (BP53-11, ready to use, Ventana) immunohistochemistry, with appropriate positive and negative controls, was carried out according to manufacturer’s instructions on Benchmark Ultra Immunostainer (Roche).

Defined areas adjudged to be dVIN were marked on the HE-stained slides for accurate comparison with the slides stained with immunohistochemistry. Immunohistochemistry slides were scored by SDG, PEG and SK on a multi-head microscope. The stains were analysed as described below.

#### CK13 and CK17

The percentage of cells showing cytoplasmic staining; the intensity of staining (weak, moderate, and strong) and their distribution in the epithelium was noted. Receiver operating characteristic (ROC) curves were plotted for CK13 and CK17, using the percentage of cell stained, to assess their individual sensitivity and specificity for dVIN diagnosis. In order to assess whether these markers perform better when interpreted together, another ROC curve was derived.

#### MIB1

Increased MIB1 expression was noted as more than sporadic nuclear staining in the basal and/or suprabasal layers.

#### p53

Staining with p53 was recorded as overexpression, null pattern or wild type. Intense nuclear staining in ≥ 50% cells in the lower one third of the epithelium, occasionally extending to the suprabasal layers was considered overexpression. Nuclear staining of weak to moderate intensity, in < 50% cells in the lower one third of epithelium was noted as wild type. Complete absence of staining was noted as null pattern.

### Statistical analysis

Data analysis was done with IBM SPSS Statistics 24 (SPSS, Chicago, IL, USA). Independent sample’s *t* test was used for parametric data and chi-square (*χ*^2^) test for non-parametric data to deduce the *p* value. A *p* value < 0.05 was considered statistically significant. Interobserver agreement was measured with Cohen’s kappa. Kappa (*κ*) was interpreted as < 0.20 = poor, 0.21–0.40 = fair, 0.41–0.60 = moderate, 0.61–0.80 = substantial and 0.81–1.00 = almost perfect agreement.

## Results

From the archives, 180 cases of dVIN, 105 cases of LS and 126 cases of NNED were identified. Of the 180 cases of dVIN, 61 were isolated dVIN and 119 were identified next to VSCC. Fifty-nine percent (36/61) of the isolated dVIN had a history of VSCC. For histological evaluation, 180 cases of dVIN and 105 cases of LS were included.

### Histological evaluation

#### Nuclear atypia

All cases of dVIN showed nuclear atypia, albeit in varying extents. Some variation in nuclear size and shape could be noticed under low magnification (×100) in 63% (114/180) of dVIN. Abnormalities of nuclear chromatin (hyperchromatic or open chromatin) were present in all dVIN cases. Angulated nuclei, seen in 66% (119/180) of dVIN, and macronucleoli, seen in 65% (118/180) of dVIN had the strongest statistical significance (*p* < 0.001).

#### Mitoses

Atypical mitoses and suprabasal mitoses were noted more frequently than a mitotic count > 5/5 mm in dVIN.

#### Disturbed maturation

Individual cell keratinisation was present in 92% (165/180), deep keratinisation in 78% (141/180) and deep squamous eddies in 61% (110/180) of dVIN cases. Cobblestone appearance of the epithelium was noted in 83% (149/180) of dVIN cases. All of these features had strong statistical significance for dVIN (*p* < 0.001).

#### Architecture

Elongated rete ridges were present in 63% (114/180) of dVIN (*p* < 0.01) and appeared to be anastomosing in 20% (37/180) of cases.

#### Other features

**Fig. 1:**
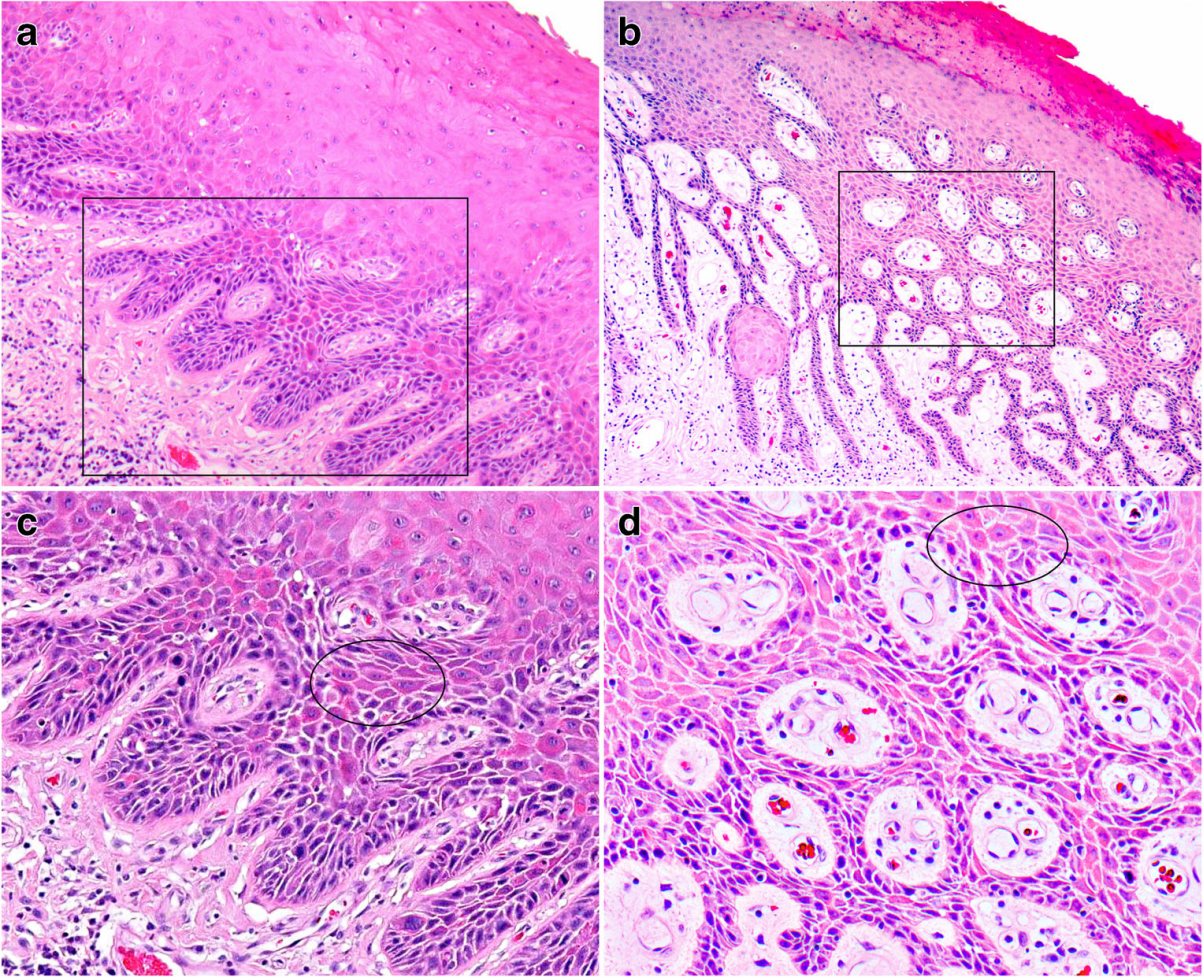
Example of differentiated VIN with characteristic features (HE stain), low magnification appearance (**a**, **b**), with corresponding higher magnification images (**c**, **d**). **a** A widened epithelium with parakeratosis and elongated rete ridges is seen. Nuclear atypia, premature keratinisation and cobblestone appearance are apparent (original magnification ×50). **b** Elongated rete ridges, a deep squamous eddy, nuclear atypia, and parakeratosis can be identified under low magnification (original magnification ×50). **c** Under higher magnification, macronucleoli can be seen. Angulated nuclei, individual cell keratinisation and cobblestone appearance (circled area) can be better appreciated (original magnification ×100). **d** Atypical cells with both open chromatin and hyperchromatic patterns are seen. There is cobblestone appearance (circled area) and individual cell keratinisation (original magnification ×100)

**Fig. 2:**
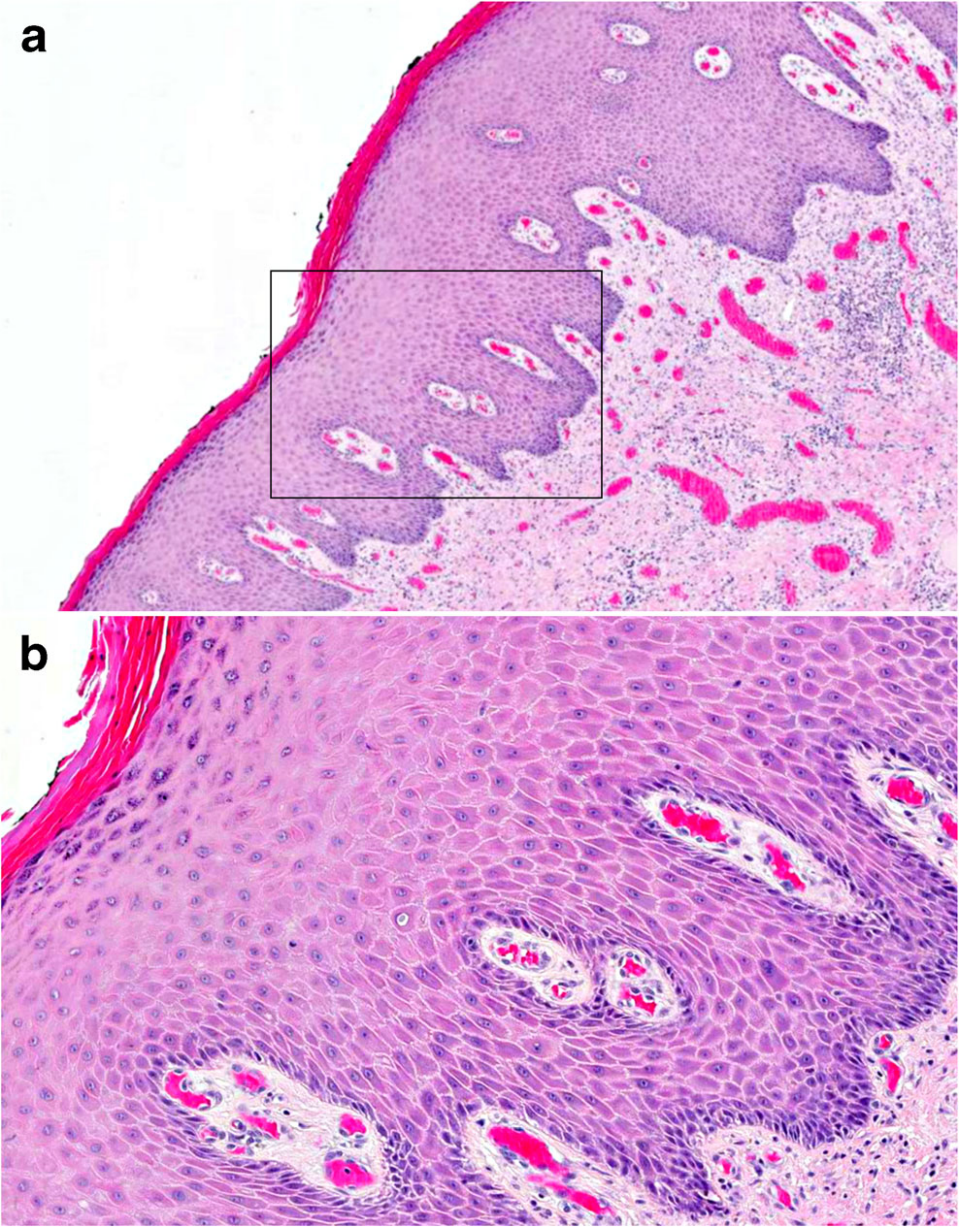
Example of differentiated VIN with relatively subtle histological features (HE stain). **a** Nuclear atypia cannot be easily discerned under low magnification (in contrast to Fig. 1a, c). A widened epithelium, parakeratosis, some elongation of rete ridges and mildly increased cellularity are seen (original magnification ×50). **b** Under higher magnification, nuclear atypia with open chromatin pattern, macronucleoli, individual cell keratinisation and cobblestone appearance are detectable (original magnification ×100)

Parakeratosis was noted in 73% (132/180) of dVIN (*p* < 0.01). An overview of the histological evaluation is given in Table 1, and the histological features are illustrated in Figs. 1 and 2.

**Table 1:** Evaluation of histological features (dVIN vs LS)

Histological features	dVIN (*n* = 180)	LS (*n* = 105)	*p* value (dVIN vs LS)
	Number (percentage)	Number (percentage)	
A. Nuclear atypia			
Obvious under low power (×100)	114 (63)	0	0.01
Angulated nuclei	119 (66)	8 (8)	0.001
Chromatin pattern			
- Open	96 (53)	6 (6)	0.01
- Hyperchromatic	84 (47)	10 (10)	0.01
Macronucleoli	118 (65)	24 (23)	0.001
Multinucleation	129 (72)	65 (62)	0.20
B. Mitotic figures			
Mitotic count > 5/5 mm	67 (37)	20 (19)	0.003
Atypical mitoses	102 (57)	0	< 0.001
Suprabasal mitoses	122 (68)	13 (12)	< 0.01
C. Disturbed maturation			
Individual cell keratinisation	165 (92)	21 (20)	< 0.001
Deep keratinisation	141 (78)	4 (4)	< 0.001
Deep eddies	110 (61)	0	< 0.001
Cobblestone appearance	149 (83)	12 (11)	0.001
D. Architecture			
Elongated rete ridges	114 (63)	7 (7)	0.01
- Elongated and anastomosing	37 (21)	0	0.01
E. Others			
Inflammatory response			
- Scanty/focal	41 (23)	17 (16)	
- Moderate	82 (46)	51 (49)	0.25
- Marked	57 (32)	37 (35)	
Sub-epithelial hyalinisation	124 (69)	81(77)	0.10
Hyperkeratosis	125 (69)	77 (73)	0.58
Parakeratosis	132 (73)	56 (53)	0.009

### Reproducibility

We found ‘substantial’ interobserver agreement (*κ* = 0.71, standard error (SE) = 0.11, 95% confidence interval (CI) 0.51–0.95) on the selected subset of cases for the diagnosis of dVIN. Out of all the histological features evaluated from the checklist, substantial agreement was obtained for macronucleoli (*κ* = 0.75), deep keratinisation (*κ* = 0.71), deep squamous eddies (*ĸ* = 0.68), individual cell keratinisation (*κ* = 0.66), mitotic count > 5/5 mm (*κ* = 0.64) and angulated nuclei (*ĸ* = 0.60). The histological features with substantial and ‘moderate’ agreement are listed in Table 1 of the Supplementary Material.

### Immunohistochemistry

Immunohistochemistry with p53, CK13 and dual-stain CK17-MIB1 was conducted on an initial set of 24 cases of dVIN, 9 cases of LS and 8 cases of NNED. For further evaluation of CK13 and CK17, these stains were carried out on an additional set comprising 30 cases of dVIN, 5 cases of LS and 22 cases of NNED.

#### Cytokeratin 13

**Fig. 3:**
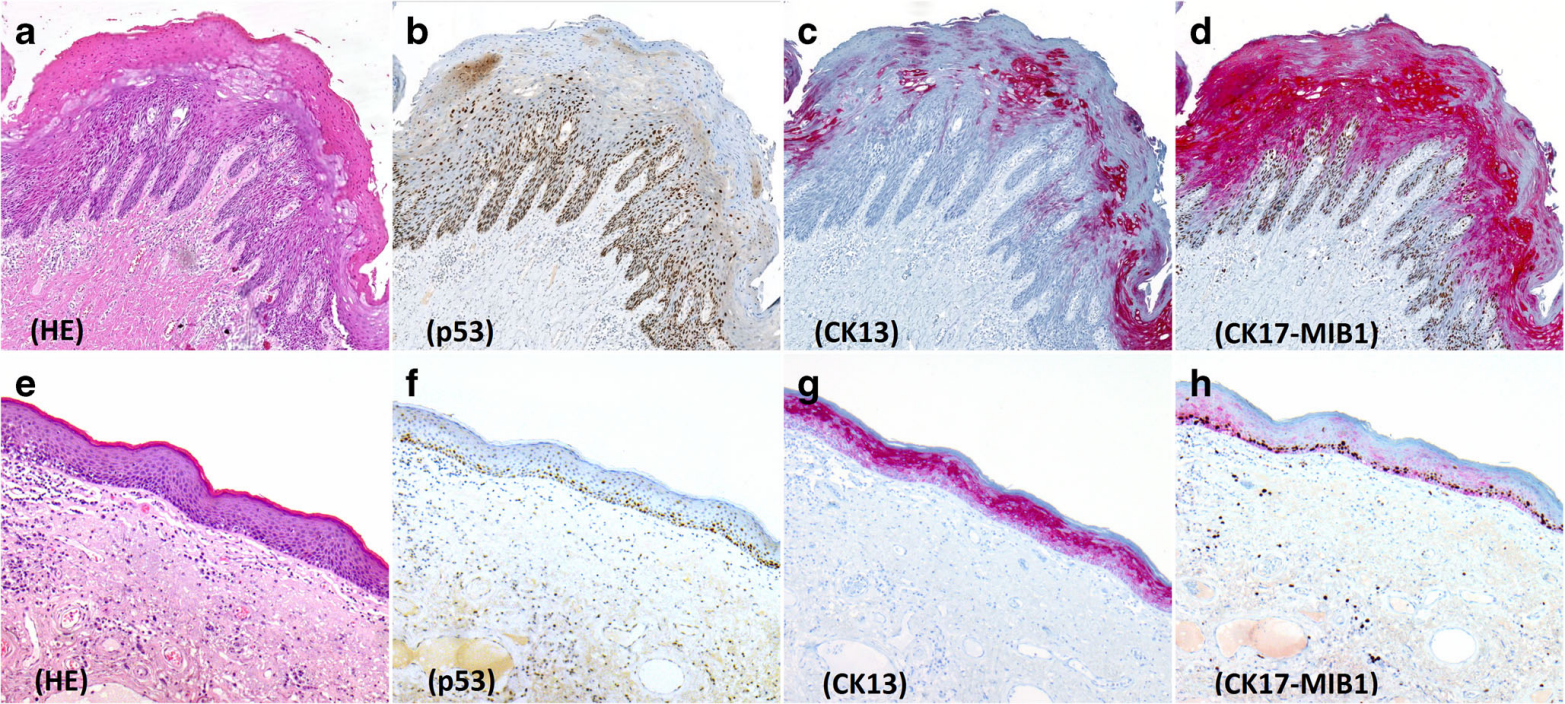
Immunohistochemistry in differentiated VIN (**a–d**) and Lichen sclerosus (**e–h**). **a** Differentiated VIN, HE stain **b** Overexpression of p53. **c** Weak, patchy CK13 staining. **d** Strong and diffuse CK17 expression, with increased MIB-1. **e** Lichen sclerosus, HE stain. **f** Wild-type p53 expression. **g** Diffuse staining of moderate intensity with CK13. **h** Very weak, patchy CK17 staining with increased MIB-1

For LS and other NNED cases, the percentage of cells staining with CK13 was higher compared to dVIN, and the staining intensity was stronger (Fig. 3).

##### Differentiated VIN (*n* = 54)

In 15% (8/54) of dVIN, there was a complete lack of CK13 staining. Patchy, weak staining in the suprabasal layers was seen in 67% (36/54); and diffuse, weak staining in the superficial layers was seen in 18% (10/54). The mean percentage of cells positive for CK13 was 15 (95% CI = 9.4–20.5).

##### Lichen sclerosus (*n* = 14)

None of the LS cases showed complete lack of CK13 staining. In 79% (11/14), diffuse staining of moderate intensity was noted in the suprabasal layers; and in 21% (3/14), patchy staining of moderate intensity was noted in the suprabasal layers. The mean percentage of cells positive for CK13 was 31 (95% CI = 11.4–50).

##### NNED (*n* = 30)

Complete lack of CK13 staining was also not seen in any of the NNED cases. In 57% (17/30) of cases, diffuse staining of moderate intensity was noted in the suprabasal layers; in 43% (13/30), patchy staining of moderate intensity was noted in the suprabasal layers. The mean percentage of cells positive for CK13 was 39 (95% CI = 23.6–53.8).

#### Cytokeratin 17

For dVIN, the percentage of cells staining with CK17 was higher compared to LS and other NNED cases, and staining intensity was stronger (Fig. 3).

##### Differentiated VIN (*n* = 54)

Diffuse, strong staining, across the full thickness of the epithelium was seen in 33% (18/54). The rest of the dVIN showed diffuse, moderate to strong staining in the suprabasal layers in 56% (30/54) of cases, and patchy, strong staining in the superficial layers in 11% (6/54) of cases. The mean percentage of cells positive for CK17 was 74 (95% CI = 68.5–81.2).

##### Lichen sclerosus (*n* = 14)

None of the cases of LS showed diffuse, strong staining across full epithelial thickness. Patchy, weak staining in the suprabasal layers was seen in 64% (9/14), and diffuse, weak staining in the superficial layers in 36% (5/14). The mean percentage of cells positive for CK17 was 41 (95% CI = 18.4–51.6).

##### NNED (*n* = 30)

Complete absence of CK17 staining was noted in 57% (17/30) of NNED cases. Patchy, weak staining in the suprabasal layers was seen in 27% (8/30), and diffuse, weak staining in the superficial layers was seen in 16% (5/30). The mean percentage of cells positive for CK17 was 19 (95% CI = 7.8–31.1).

**Fig. 4:**
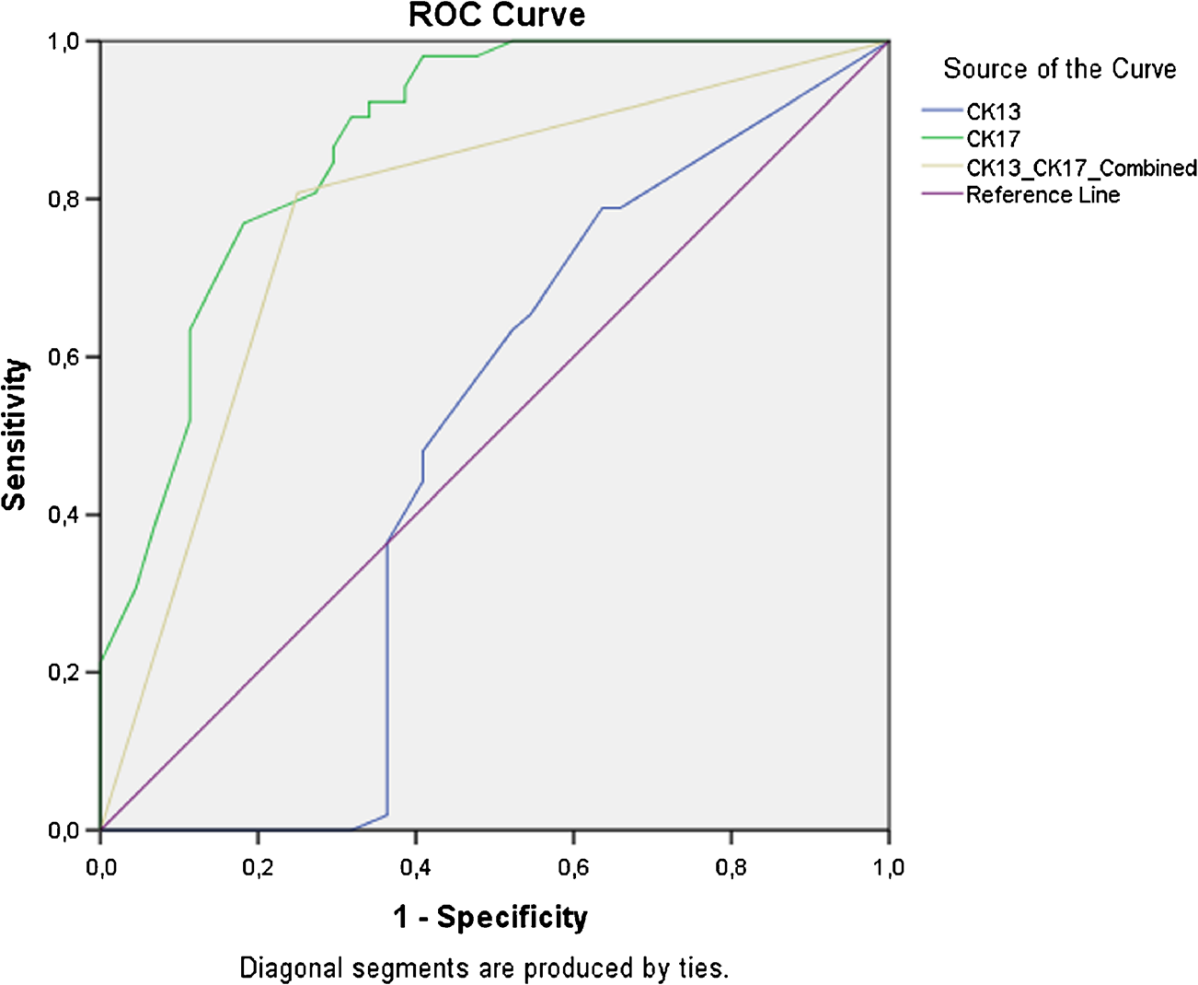
ROC curves for CK13 and CK17 immunohistochemistry for the diagnosis of dVIN. The green line represents CK17 and the blue line represents CK13, when they are interpreted individually. The yellow line represents the ROC curve when CK13 and CK17 are interpreted together. Area under the curve (AUC) for CK13 = 0.52, CK17 = 0.87 and CK13 and CK17 combined = 0.76

On computing ROC curves, CK13 had an area under the curve (AUC) of 0.52 (SE 0.06, 95% CI = 0.40–0.64), while CK17 had an AUC of 0.87 (SE 0.04, 95% CI = 0.80–0.94). The combination of both stains, i.e. when CK13 and CK17 immunohistochemistry was interpreted together, showed an AUC of 0.76, (SE 0.05, 95% CI = 0.70–0.87) (Fig. 4).

#### MIB1

Increased MIB1 was noted in 67% (36/54) dVIN, 36% (5/14) LS, and 47% (14/30) other NNED cases; the details are elaborated in Table 2 of the Supplementary Material.

#### p53

##### Differentiated VIN (*n* = 24)

Overexpression of p53 was seen in 45% (11/24), null pattern in 13% (3/24), and wild-type expression in 42% (10/24). Cases with null pattern and overexpression of p53 were considered positive for dVIN (Fig. 3).

##### Lichen sclerosus (*n* = 9)

None of the cases of LS showed the ‘null pattern’. Overexpression of p53 was seen in 33% (3/9) and wild-type expression in 67% (6/9) of LS cases (Fig. 3).

##### NNED (*n* = 8)

All the NNED cases had wild-type p53 expression. The details of p53 expression are elaborated in Table 3 of the Supplementary Material.

## Discussion

Differentiated VIN was first recognised as a precursor lesion of VSCC in 1961 [2]. Over the years, a plethora of descriptive terminology (e.g. vulvar dystrophy/hyperplasia with atypia) led to incorrect categorisation of this lesion. Recent studies report that the non-HPV related pathway contributes to around 80% of all VSCC [1, 2]. However, dVIN comprises only 2–29% of standalone VIN diagnoses [2, 18–20]. This implies that dVIN may be under-recognised, underlining the need for well-defined diagnostic criteria.

Clinically, dVIN is known to present with vague grey-white discoloration [2–5], on the background of long-standing LS, and shows subtle histological features, which are often difficult to distinguish from LS. However, dVIN is known to progress rapidly to invasive carcinoma, with a reported median interval of 28 months [5, 18, 21]. Thus, dVIN is often only recognised on histology adjacent to VSCC or on follow-up biopsies. Literature describes the presence of dVIN next to VSCC in up to 40% of cases [21]. In our study, dVIN was identified next to 49% of VSCC cases.

To facilitate the reliable diagnosis of dVIN, we set out to quantify its individual histological features. We found that nuclear atypia, the sine qua non for dVIN diagnosis, could be discerned under low power in only 63% of cases. Amongst the components of nuclear atypia, macronucleoli and angulated nuclei were the most specific. Both these features had substantial interobserver agreement in terms of relevance for a dVIN diagnosis. They can therefore be useful to discriminate the nuclear atypia of dVIN from the reactive nuclear enlargement seen in LS and other NNED. Abnormality of the nuclear chromatin was noted in all dVIN cases, with hyperchromatic or an open chromatin pattern occurring with almost equal frequency. A mitotic count of > 5/5 mm and atypical mitoses, although specific for dVIN, were seen less frequently. Multinucleation, a common feature of dVIN, was also seen regularly in LS and other NNED cases and thus lacked statistical significance for dVIN diagnosis.

Disturbed maturation in the form of premature keratinisation in the basal or parabasal layers is a morphological reflection of the underlying pathology. Manifestations of disturbed maturation (individual cell keratinisation, deep keratinisation and deep eddies) were commonly noted in dVIN. The features of disturbed maturation had the second highest level of agreement amongst our pathologists, next in importance only to macronucleoli for dVIN diagnosis. Cobblestone appearance of the epithelium [22], elongated (± anastomosing) rete ridges and parakeratosis could also be reproducibly identified, and these features should be regarded as important pointers towards the diagnosis of dVIN, especially in cases where nuclear atypia cannot be easily discerned. Spongiotic changes of the epithelium seen in NNED should not be mistaken for the cobblestone appearance, as the latter is always accompanied by evidence of disturbed maturation. We found individual cell keratinisation, deep keratinisation, cobblestone appearance and parakeratosis to occur more frequently than suprabasal mitosis or abnormal mitotic figures in dVIN. With this study, we hope to highlight the importance of detailed scrutiny of these supporting features.

We noticed that dVIN can show a whole spectrum of morphological features. However, even in the most subtle cases with minimal nuclear atypia, alteration of cellularity and alignment of nuclei with individual cell keratinisation and parakeratosis are present. A link between the particulars of the histological appearance and progression to VSCC could potentially be explored. This detailed description and quantification of the morphological features is primarily intended to guide the general pathologist to recognise dVIN, particularly in the dubious cases where the difference from LS may not be apparent.

Immunohistochemistry with p53 and MIB1 is often used to support the diagnosis of dVIN. However, no universal cutoff exist for the interpretation of these stains, and thus, the distinction between wild-type p53 expression and p53 overexpression may not be easy to make. We found the p53 null pattern to be specific for dVIN, but only a minority of dVIN show this pattern. A proportion of both dVIN and LS showed wild-type expression and utility of p53 can be limited in these cases.

The second step of this study was to evaluate CK13 and CK17 as potential diagnostic adjuncts for dVIN. Cytokeratins are cell type-specific intermediate filament proteins and their expression is altered in abnormalities of cellular differentiation. Expression patterns of cytokeratins 8, 10, 13 and 14 in VSCC were studied in 1995 by Ansink et al. [23]. They noted CK13 expression in well-differentiated VSCC, as well as in the normal epithelium of labium minus. Recently, there has been a lot of interest in cytokeratin research, particularly for (high-grade) oral dysplasia.

Cytokeratin 13 is expressed physiologically from the prickle cell layer (third basal layer) to the keratinised layer (surface) in normal oral mucosa [24, 25]. Progressive loss of CK13 with increasing grades of dysplasia has been demonstrated in the oral cavity, cervix and oesophagus [25–32]. Cytokeratin 17 is a basal/myoepithelial cell keratin which is not expressed under physiological conditions in oral mucosa or perianal skin [16, 33, 34]. Increased CK17 expression has been reported in oral, cervical and anal intraepithelial neoplasia [17, 31, 33, 34]. There is little information about CK17 expression in normal vulvar tissue. In dVIN, increased CK17 expression has been reported on in a single study [14].

We found CK13 expression to be lower in dVIN compared to LS and other NNED. On the other hand, CK17 expression was higher in dVIN than in LS and other NNED. From analysing the ROC curves, increased CK17 expression showed better sensitivity and specificity than CK13 loss for dVIN. Similar to the findings of Podoll et al. [14], diffuse CK17 staining across full epithelial thickness or in the suprabasal layers was found to be strongly supportive of a dVIN diagnosis. Complete lack of CK13 staining was specific for dVIN, but this occurred in only 15% of cases. In some cases, CK17 immunohistochemistry may be equivocal, for example, patchy, strong staining or diffuse staining in the superficial layers only. In this situation, a reduced expression or a complete lack of CK13 staining can offer additional support for the diagnosis of dVIN.

Our study, in common with most retrospective studies, has some limitations; a selection bias cannot be ruled out. For testing the reproducibility, a limited number of cases selected by two pathologists was included, and the two other participants were experienced pathologists from the same institute. Thus, our results may not entirely reflect daily diagnostic practice. External validation studies, with more cases and participants from other centres will follow. With respect to immunohistochemistry, more extensive research on the expression of CK17 in vulvar skin and mucosa is necessary to establish its relevance in practice.

Despite the limitations, we have attempted to describe here the most helpful histological features to enable the diagnosis of dVIN. Increased CK17 expression has potential as a diagnostic adjunct for dVIN and deserves further exploration in this context.

## Conclusion

Macronucleoli and angulated nuclei should alert the pathologist to consider the diagnosis of dVIN. Disturbed maturation and cobblestone appearance are other specific and reproducible features of dVIN and may be of particular use where nuclear atypia is less prominent. Increased CK17 expression may have promise as an adjunct to histology for discriminating dVIN from close differentials.

## Electronic supplementary material

ESM 1 (DOCX 16 kb)

## References

[1] WHO (2014). Classification of tumours of female reproductive organs.

[2] Hoang LN, Park KJ, Soslow RA, Murali R (2016). Squamous precursor lesions of the vulva: current classification and diagnostic challenges. Pathology.

[3] Medeiros F, Nascimento AF, Crum CP (2005). Early vulvar squamous neoplasia advances in classification, diagnosis, and differential diagnosis. Adv Anat Pathol.

[4] van den Einden LCG, de Hullu JA, Massuger LF (2013). Interobserver variability and the effect of education in the histopathological diagnosis of differentiated vulvar intraepithelial neoplasia. Mod Pathol.

[5] Yang B, Hart WR (2000). Vulvar intraepithelial neoplasia of the simplex (differentiated) type: a clinicopathologic study including analysis of HPV and p53 expression. Am J Surg Pathol.

[6] Reutter JC, Walters RA, Selim MA (2016). Differentiated vulvar intraepithelial neoplasia: what criteria do we use in practice?. J Low Genit Tract Dis.

[7] van de Nieuwenhof HP, van der Avoort IAM, de Hullu JA (2008). Review of squamous premalignant vulvar lesions. Crit Rev Oncol Hematol.

[8] Singh N, Leen SL, Han G, Faruqi A, Kokka F, Rosenthal A, Jiang XR, Kim R, McAlpine JN, Gilks CB (2015). Expanding the morphologic spectrum of differentiated VIN (dVIN) through detailed mapping of cases with p53 loss. Am J Surg Pathol.

[9] Santos M, Montagut C, Mellado B, García Á, Cajal SR, Cardesa A, Puig-Tintoré LM, Ordi J (2004). Immunohistochemical staining for p16 and p53 in premalignant and malignant epithelial lesions of the vulva. Int J Gynecol Pathol.

[10] Hantschmann P, Sterzer S, Jeschke U, Friese K (2005). p53 expression in vulvar carcinoma, vulvar intraepithelial neoplasia, squamous cell hyperplasia and lichen sclerosus. Anticancer Res.

[11] Gambichler T, Kammann S, Tigges C, Kobus S, Skrygan M, Meier JJ, Köhler CU, Scola N, Stücker M, Bechara FG, Altmeyer P, Kreuter A (2011). Cell cycle regulation and proliferation in lichen sclerosus. Regul Pept.

[12] Rolfe KJ, Eva LJ, MacLean AB, Crow JC, Perrett CW, Reid WM (2001). Cell cycle proteins as molecular markers of malignant change in vulvar lichen sclerosus. Int J Gynecol Cancer.

[13] Liegl B, Regauer S (2006). p53 immunostaining in lichen sclerosus is related to ischaemic stress and is not a marker of differentiated vulvar intraepithelial neoplasia (d-VIN). Histopathology.

[14] Podoll MB, Singh N, Gilks CB, Moghadamfalahi M, Sanders MA (2016). Assessment of CK17 as a marker for the diagnosis of differentiated vulvar intraepithelial neoplasia. Int J Gynecol Pathol.

[15] Mikami T, Cheng J, Maruyama S, Kobayashi T, Funayama A, Yamazaki M, Adeola HA, Wu L, Shingaki S, Saito C, Saku T (2011). Emergence of keratin 17 vs. loss of keratin 13: their reciprocal immunohistochemical profiles in oral carcinoma in situ. Oral Oncol.

[16] Kitamura R, Toyoshima T, Tanaka H, Kawano S, Kiyosue T, Matsubara R, Goto Y, Hirano M, Oobu K, Nakamura S (2012). Association of cytokeratin 17 expression with differentiation in oral squamous cell carcinoma. J Cancer Res Clin Oncol.

[17] Yagyuu T, Obayashi C, Ueyama Y, Takano M, Tanaka Y, Kawaguchi M, Takeda M, Kasai T, Kirita T (2015). Multivariate analyses of Ki-67, cytokeratin 13 and cytokeratin 17 in diagnosis and prognosis of oral precancerous lesions. Oral Pathol Med.

[18] van de Nieuwenhof HP, Bulten J, Hollema H, Dommerholt RG, Massuger LFAG, van der Zee AGJ, de Hullu JA, van Kempen LCLT (2011). Differentiated vulvar intraepithelial neoplasia is often found in lesions, previously diagnosed as lichen sclerosus, which have progressed to vulvar squamous cell carcinoma. Mod Pathol.

[19] Scurry J, Campion M, Scurry B, Kim SN, Hacker N (2006). Pathologic audit of 164 consecutive cases of vulvar intraepithelial neoplasia. Int J Gynecol Pathol.

[20] Eva LJ, Ganesan R, Chan KK, Honest H, Luesley DM (2009). Differentiated-type vulval intraepithelial neoplasia has a high-risk association with vulval squamous cell carcinoma. Int J Gynecol Cancer.

[21] van de Nieuwenhof HP, Massuger LF, van der Avoort IA (2009). Vulvar squamous cell carcinoma development after diagnosis of VIN increases with age. Eur J Cancer.

[22] Wasserman JK, Bateman J, Mai KT (2016). Differentiated squamous intraepithelial neoplasia associated with squamous cell carcinoma of the anal canal. Histopathology.

[23] Ansink A, Mooi WJ, van Doorneward G, van Tinteren H, Heintz APM, Ivanyi D (1995). Cytokeratin subtypes and Involucrin in squamous cell carcinoma of the vulva; an immunohistochemical study of 41 cases. Cancer.

[24] Nobusawa A, Sano T, Negishi A, Yokoo S, Oyama T (2014). Immunohistochemical staining patterns of cytokeratins 13, 14, and 17 in oral epithelial dysplasia including orthokeratotic dysplasia. Pathol Int.

[25] Ida-Yonemochi H, Maruyama S, Kobayashi T, Yamazaki M, Cheng J, Saku T (2012). Loss of keratin 13 in oral carcinoma in situ: a comparative study of protein and gene expression levels using paraffin sections. Mod Pathol.

[26] Yamashina M, Sato K, Tonogi M, Tanaka Y, Yamane GY, Katakura A (2014). Evaluation of superficial oral squamous cell malignancy based on morphometry and immunoexpression of cytokeratin 13 and cytokeratin 17. Acta Cytol.

[27] Ohkura S, Kondoh N, Hada A, Arai M, Yamazaki Y, Sindoh M, Takahashi M, Matsumoto I, Yamamoto M (2005). Differential expression of the keratin-4, -13, -14, -17 and transglutaminase 3 genes during the development of oral squamous cell carcinoma from leukoplakia. Oral Oncol.

[28] Bloor BK, Seddon SV, Morgan PR (2001). Gene expression of differentiation-specific keratins in oral epithelial dysplasia and squamous cell carcinoma. Oral Oncol.

[29] Schaaij-Visser TB, Bremmer JF, Braakhuis BJ (2010). Evaluation of cornulin, keratin 4, keratin 13 expression and grade of dysplasia for predicting malignant progression of oral leukoplakia. Oral Oncol.

[30] Noguchi S, Sato K, Yamamoto G, Tonogi M, Tanaka Y, Tachikawa T, Yamane GY (2011). Expression of cytokeratin 13 and 17 in tongue squamous cell carcinoma and epithelial dysplasia. Asian J Oral Maxillofac Surg.

[31] Carrilho C, Alberto M, Buane L, David L (2004). Keratins 8, 10, 13, and 17 are useful markers in the diagnosis of human cervix carcinomas. Hum Pathol.

[32] Takahashi H, Shikata N, Senzaki H, Shintaku M, Tsubura A (1995). Immunohistochemical staining patterns of keratins in normal oesophageal epithelium and carcinoma of the oesophagus. Histopathology.

[33] Khanom R, Nguyen CTK, Kayamori K, Zhao X, Morita K, Miki Y, Katsube KI, Yamaguchi A, Sakamoto K (2016). Keratin 17 is induced in oral cancer and facilitates tumor growth. PLoS One.

[34] Nazarian RM, Primiani A, Doyle LA, Linskey KR, Duncan LM, Odze RD, Zukerberg LR (2014). Cytokeratin 17: an adjunctive marker of invasion in squamous neoplastic lesions of the anus. Am J Surg Pathol.

