# Supplementary material for: Differentiated vulvar intraepithelial neoplasia (dVIN): the most helpful histological features and the utility of cytokeratins 13 and 17
Source: Virchows Arch. 2018 Sep 6;473(6):739–47. doi: 10.1007/s00428-018-2436-8 (PMC6267258; doi:10.1007/s00428-018-2436-8)
Supplement: Supplementary file 1 — (DOCX 16 kb) [file 428_2018_2436_MOESM1_ESM.docx]

# Supplementary material

# Table 1: Kappa value for the histological features that showed substantial and moderate agreement ^a^

| **Histological features** | **Kappa (κ)** | **Agreement** | **SE ^b^** | **95% CI ^c^** |
| --- | --- | --- | --- | --- |
| Macronucleoli | 0.75 | Substantial | 0.14 | 0.49 - 1 |
| Deep keratinisation | 0.71 | Substantial | 0.13 | 0.47 - 1 |
| Deep squamous eddies | 0.68 | Substantial | 0.11 | 0.46 - 0.9 |
| Individual cell keratinisation | 0.66 | Substantial | 0.26 | 0.12 - 1 |
| Mitotic count >5/5mm | 0.64 | Substantial | 0.32 | 0.02 - 1 |
| Angulated nuclei | 0.60 | Substantial | 0.33 | 0.02 - 1 |
| Cobblestone appearance | 0.56 | Moderate | 0.14 | 0.26 - 0.8 |
| Elongated ± anastomosing rete ridges | 0.51 | Moderate | 0.18 | 0.16 - 0.86 |
| Atypical mitoses | 0.44 | Moderate | 0.22 | 0.02 - 0.88 |

^a^ See Table 1 of manuscript for the complete list of histological features; ^b^ SE: Standard Error, ^c^ CI: Confidence Interval

## Table 2: MIB1 immunohistochemistry in dVIN, LS, and other NNED

| **MIB1** | **Vulvar lesions**  **Number of cases (percentage)** | | |
| --- | --- | --- | --- |
| Increased | **dVIN (n = 54)** | **LS (n = 14)** | **Other NNED (n = 30)** |
|  | 36 (67) | 5 (36) | 14 (47) |
| Not increased | 18 (33) | 9 (64) | 16 (53) |

## Table 3: p53 immunohistochemistry in dVIN, LS, and other NNED

| **Staining patterns of p53** | **Vulvar lesions**  **Number of cases (percentage)** | | |
| --- | --- | --- | --- |
|  | **dVIN (n = 24)** | **LS (n = 9)** | **Other NNED (n = 8)** |
| **Null pattern** | 3 (13) | - | - |
| **Wild type** | 10 (42) | 6 (67) | 8 (100) |
| **Overexpression** | 11 (45) | 3 (33) | - |
